# Downregulation of Lonp1 Promotes Melanocyte Pyroptosis via Suppressing Mitophagy and Activating NLRP3 Pathway in Vitiligo under Oxidative Stress

**DOI:** 10.1007/s10753-026-02538-y

**Published:** 2026-06-17

**Authors:** Xin Huang, Tianqi Wei, Youming Guo, Jing Zhu, Lingling Luo, Zhaokang Zhou, Baiyi Zuo, Guanying Liu, Beilei Xu, Chengrang Li

**Affiliations:** 1 Hospital for Skin Diseases, Institute of Dermatology, Chinese Academy of Medical Sciences & Peking Union Medical College, Nanjing, Jiangsu China; 2 Department of Paediatrics and Adolescent Medicine, The University of Hong Kong, Hong Kong, Hong Kong China; 3 Wuxi No.2 Peoples Hospital, Wuxi, Jiangsu China

**Keywords:** Vitiligo, Melanocytes, Mitophagy, NLRP3, IL-1β, Pyroptosis

## Abstract

Vitiligo is an autoimmune skin disease characterized by the loss of epidermal melanocytes. Oxidative stress serves as a key initiating factor in its pathogenesis. Mitochondria, known as the powerhouse of the cell, perform multiple essential functions in eukaryotic cells and participate in melanocyte physiological processes. Lonp1 is a crucial mitochondrial matrix soluble protease involved in maintaining mtDNA stability, clearing aberrant proteins, and regulating mitochondrial homeostasis. Meanwhile, mitophagy serves as a crucial function within the mitochondrial quality control system, responsible for eliminating damaged mitochondria. Pyroptosis is a form of programmed cell death mediated by inflammasomes, accompanied by cell membrane pore formation and the release of inflammatory cytokines. This study confirmed that oxidative stress was associated with decreased Lonp1 in PIG1 cells, the human melanocyte line. This downregulation impairs mitochondrial homeostasis by suppressing the expression of PINK1, a key mitophagy-related protein, ultimately leading to activation of the NLRP3 inflammasome pathway, release of IL-1β, and induction of melanocyte pyroptosis.

## Introduction

Vitiligo is a multifactorial autoimmune skin disease characterized primarily by the loss of epidermal melanocytes. Its clinical manifestations include localized or widespread depigmentation of the skin, hair, or mucous membranes. Oxidative stress is considered a significant initiating factor in the pathogenesis of vitiligo [1].

Melanin synthesis, a unique physiological function of melanocytes, is a process composed of a series of oxidation reactions that is highly dependent on mitochondrial energy production. This process is accompanied by the generation of reactive oxygen species (ROS) as byproducts [2], including the superoxide anion (O₂⁻), hydrogen peroxide (H₂O₂), and hydroxyl radicals (·OH). Consequently, melanocytes are particularly vulnerable to oxidative stress.

Normal mitochondrial function is crucial for maintaining cellular redox homeostasis and protecting cells from oxidative damage. Upon mitochondrial damage, accumulated ROS can disrupt the structural and functional integrity of melanocyte DNA, lipids, and proteins [3]. Concurrently, altered mitochondrial membrane permeability leads to the release of mitochondrial components or products, which act as damage-associated molecular patterns (DAMPs). These can be recognized by pattern recognition receptors, triggering immune responses [4], and ultimately resulting in melanocyte death. Therefore, mitochondrial function is closely linked to melanocyte survival and the pathogenesis of vitiligo [5]. A deeper investigation into the role of mitochondria in vitiligo pathogenesis will significantly advance our understanding of this disease.

Pyroptosis is a lytic and inflammatory form of programmed cell death mediated by gasdermin proteins, typically initiated by inflammasome activation. This process is characterized by cell membrane pore formation, cell swelling, and subsequent release of pro-inflammatory cytokines such as IL-1β and IL-18 [6]. Accumulating evidence has linked mitochondrial dysfunction to the activation of the NLRP3 inflammasome and pyroptosis in inflammatory and injury-related diseases [7, 8]. Recent studies have implicated pyroptosis in the pathogenesis of vitiligo, where it contributes to the hyperactivation of inflammasome and destruction of melanocytes [9].

The Lonp1 protease is a matrix-soluble mitochondrial protease, encoded by nuclear genes and highly conserved throughout evolution. Initially identified for mediating the selective degradation of misfolded, unassembled, or oxidatively damaged polypeptides in the mitochondrial matrix, it was later recognized as a multifunctional enzyme. It acts as a chaperone in the assembly of inner mitochondrial membrane protein complexes and participates in regulating mitochondrial gene expression (by binding to mtDNA and regulating its copy number) and maintaining mitochondrial genome integrity [10]. Furthermore, to sustain mitochondrial homeostasis, Lonp1 is involved in regulating mitochondrial dynamics and mitophagy, a specific, selective autophagy process that identifies and eliminates dysfunctional mitochondria [11]. The Lonp1 protein participates in regulating several critical cellular processes both inside and outside the mitochondria and plays a key role in maintaining organellar balance and responding to physiological and pathological stressors [12].

A previous study identified Lonp1 as a poor-prognosis factor in melanoma, where it plays a key role in metabolic reprogramming by remodeling OXPHOS complexes and protecting cancer cells against senescence [13]. This highlights the critical function of Lonp1 in maintaining mitochondrial homeostasis and cell survival under stress conditions. Given that mitochondrial dysfunction is a key trigger for NLRP3 inflammasome activation, we hypothesized that oxidative stress is associated with reduced Lonp1 expression in melanocytes, impairs mitochondrial function, and may consequently induce melanocyte pyroptosis.

## Results

### Lonp1 Expression is Decreased in PIG1 Cells Under Oxidative Stress

**Fig. 1:**
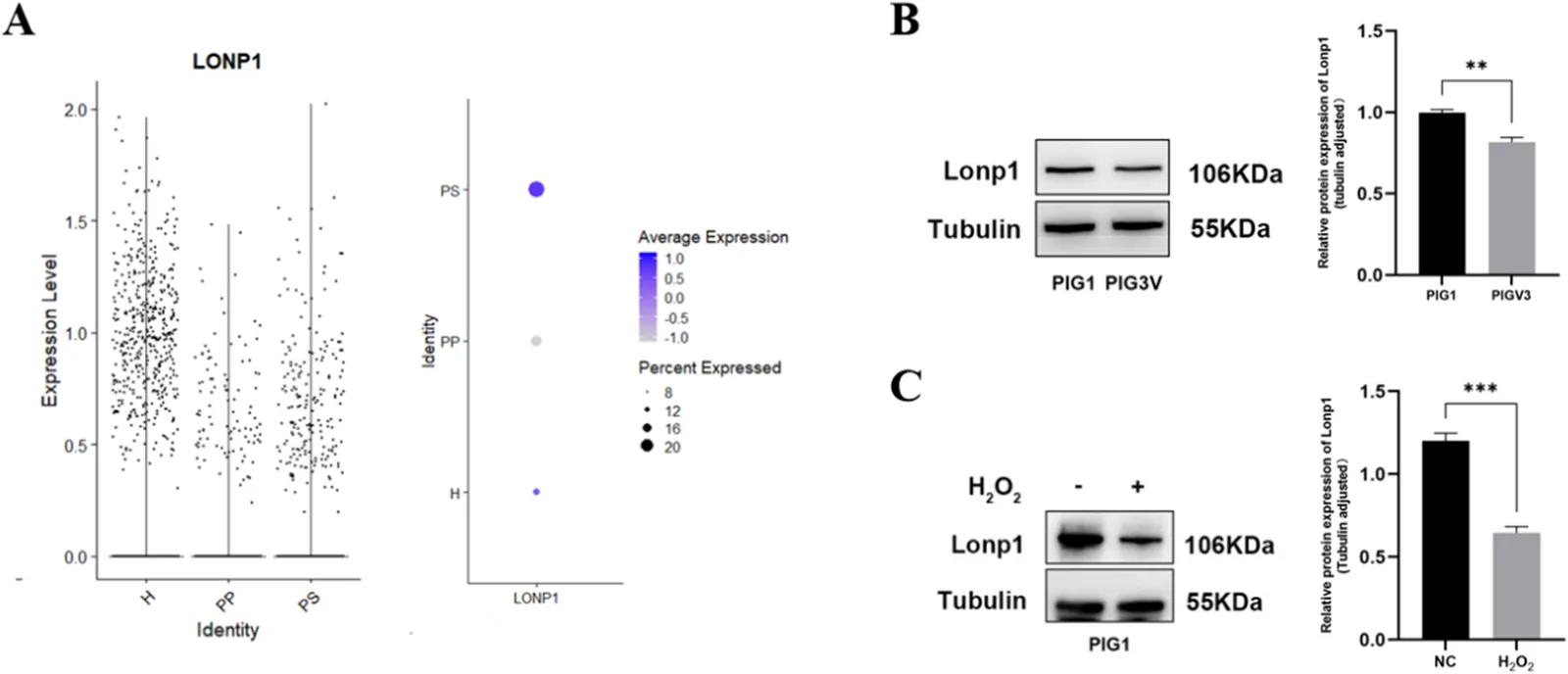
Lonp1 expression was reduced in PIG1 cells under oxidative stress. (**A**) Bioinformatic analysis of Lonp1 in single-cell sequencing data derived from skin specimens of normal individuals and vitiligo patients (Genome Sequence Archive, PRJCA006797). H, healthy (*n* = 5); PP, progressive patients (*n* = 5); PS: stable patients (*n* = 5). (**B**) Representative western blots and quantification of Lonp1 levels in PIG1 and PIG3V cells. (**C**) Representative western blots and quantification of Lonp1 levels in PIG1 cells after the treatment of 1.0 mM H_2_O_2_ for 24 h. Data are shown as mean ± SD (*n* = 3). NC, negative control. **p < 0.01, and ***p < 0.001

Firstly, we retrieved a single-cell sequencing dataset derived from skin specimens of normal individuals and vitiligo patients (Genome Sequence Archive, PRJCA006797) [14]. After unsupervised clustering, melanocytes were identified using cell markers. Following normalization, we compared molecular expression levels between different groups. We found that Lonp1 level is reduced in melanocytes from progressive vitiligo (Fig. 1A). Then, we selected PIG1 and PIG3V cell lines for this study based on their distinct origins and relevance to vitiligo research and confirmed that Lonp1 expression was decreased in PIG3V compared with PIG1 (0.82-fold; *p* = 0.0063; Fig. 1B). Oxidative stress is a key initiating factor in vitiligo. To determine the effects of oxidative stress on Lonp1 expression in melanocytes, we used H_2_O_2_ to stimulate the PIG1 cells and found that Lonp1 was downregulated (0.54-fold; *p* = 0.0007; Fig. 1C).

### Lonp1 Deficiency in PIG1 Cells was Associated with Reduced Cell Viability and Activation of the NLRP3 Inflammasome

**Fig. 2:**
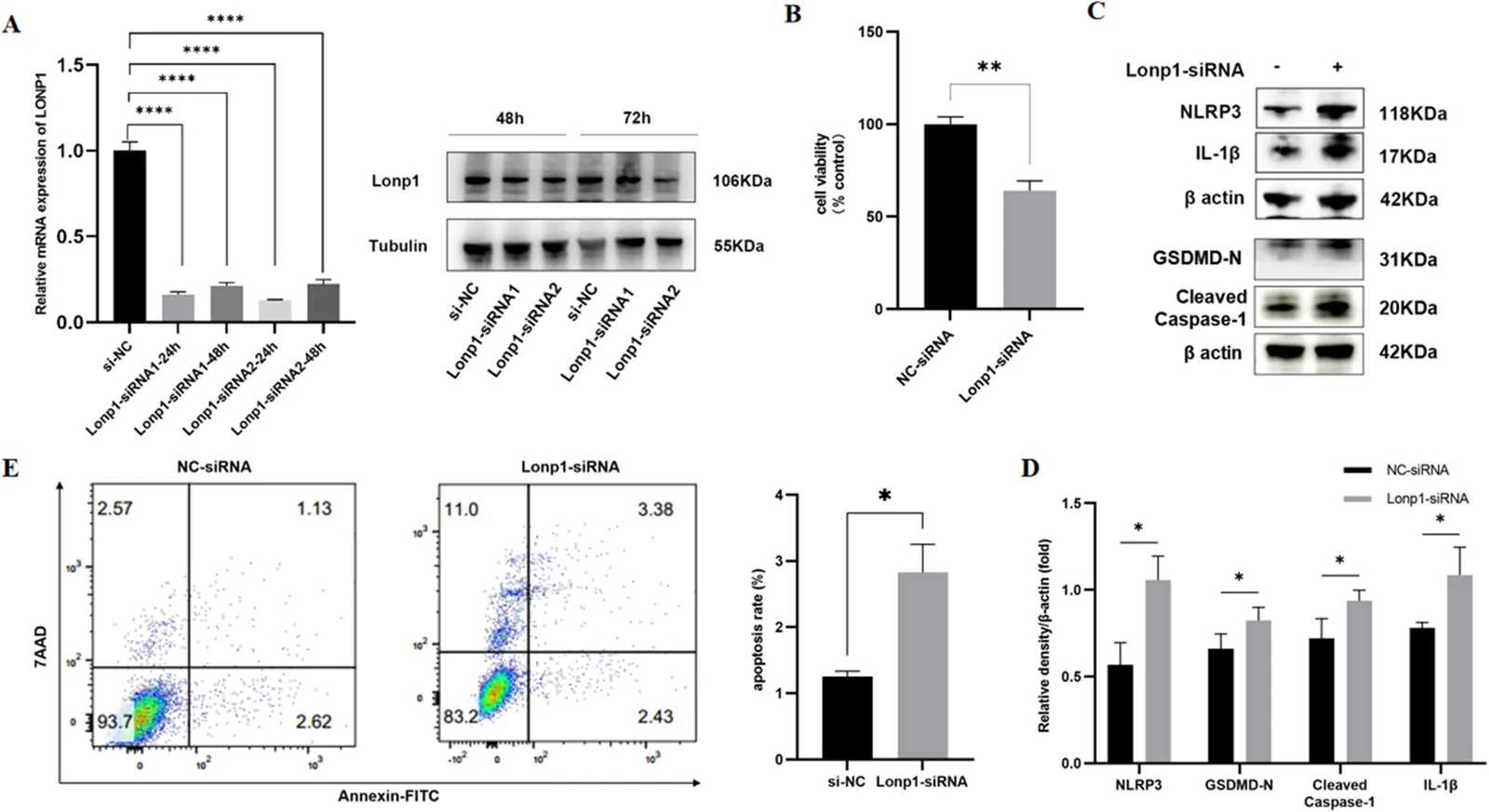
Lonp1 knockdown suppressed melanocyte activity and enhanced intracellular inflammasome activation. (**A**) Knockdown efficiency of Lonp1-siRNA in PIG1 cells assessed at the RNA level (by qRT-PCR) and protein level (by Western blot). (**B**) Cell viability was measured by CCK-8 assay in PIG1 cells transfected with NC-siRNA or Lonp1-siRNA (*n* = 3). (**C** & **D**) Representative western blots and quantification of NLPR3, GSDMD-N, Cleaved caspase-1, and IL-1β levels in PIG1 transfected with NC-siRNA or Lonp1-siRNA (*n* = 3 or 4). (**E**) Apoptosis was detected by flow cytometry following Annexin V-FITC/7-AAD staining. Annexin V labels early apoptotic cells, and 7-AAD labels late apoptotic/necrotic cells (loss of membrane integrity) (*n* = 4). NC, negative control. Data are shown as mean ± SD. *p < 0.05, **p < 0.01, and ****p < 0.0001

To explore the potential function of Lonp1 in vitiligo, we used Lonp1-specific siRNA to knock down Lonp1 in PIG1 cells, and the knockdown efficiency was confirmed by qRT-PCR and Western blot analysis (Fig. 2A). We observed that cell viability was inhibited after incubation with Lonp1-siRNA2 for 48 h (0.64-fold; *p* = 0.0055; Fig. 2B). Additionally, Annexin V-FITC/7-AAD staining revealed an increased rate of apoptosis (2.25-fold; *p* = 0.0107; Fig. 2E). Western blot analysis confirmed the upregulation of inflammasome NLRP3 (1.86-fold; *p* = 0.0108), GSDMD-N (1.25-fold; *p* = 0.0282), Cleaved caspase-1 (1.30-fold; *p* = 0.0145) and inflammatory factor IL-1β (1.39-fold; *p* = 0.0312) in Lonp1-knockdown PIG1 cells (Fig. 2C & D).

### Lonp1 Downregulation in PIG1 Cells May Regulate the NLRP3 Pathway via Suppression of the Key Mitophagy Protein PINK1

**Fig. 3:**
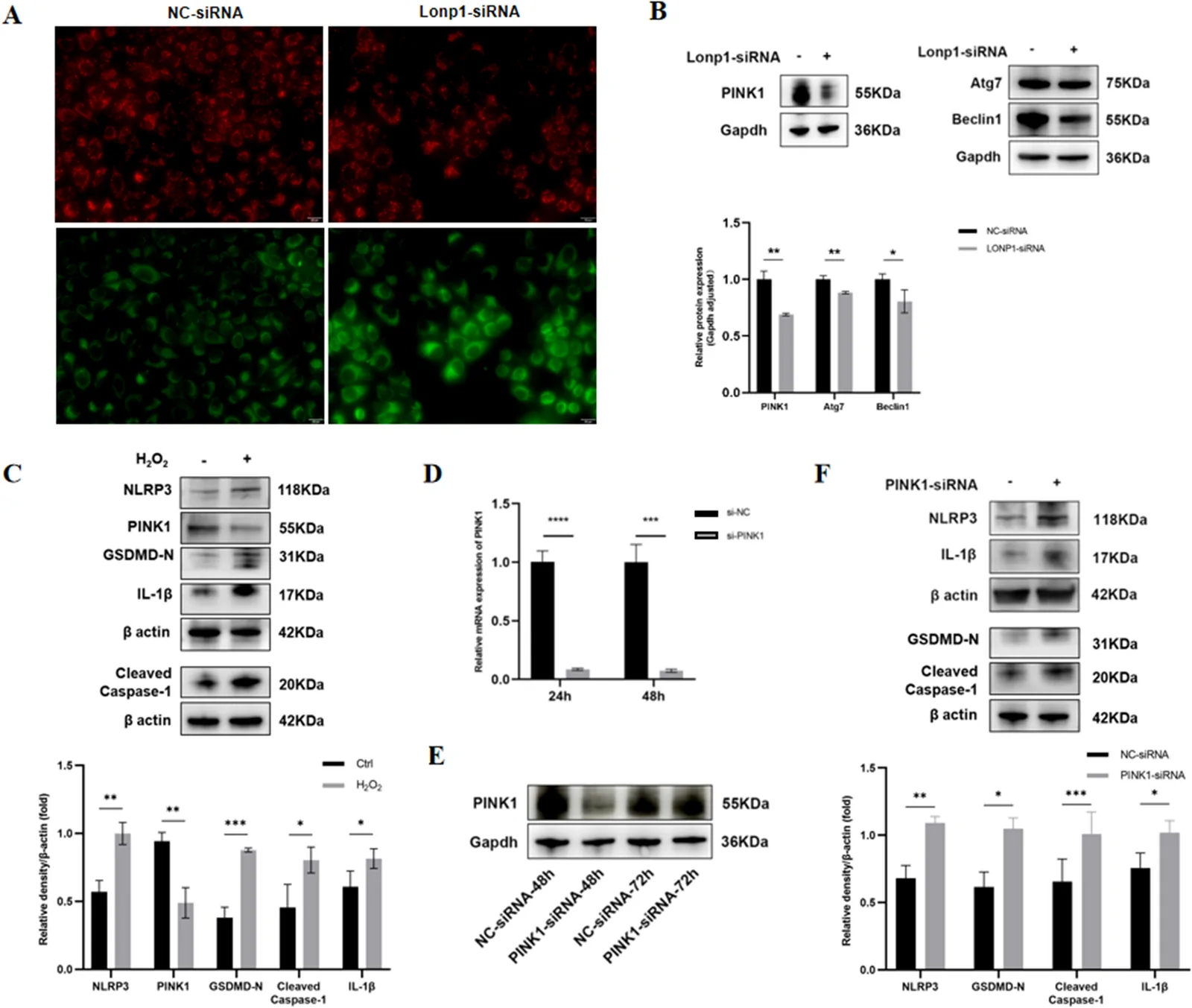
Lonp1 knockdown in melanocytes results in mitochondrial depolarization and upregulates the NLRP3 pathway via inhibiting mitophagy. (**A**) JC-1 staining was analyzed by fluorescence microscopy to evaluate mitochondrial membrane potential in PIG1 cells transfected with NC-siRNA or Lonp1-siRNA. JC-1 aggregates in polarized mitochondria emit red fluorescence, whereas JC-1 monomers in depolarized mitochondria emit green fluorescence. (**B**) Representative western blot analysis and quantification of PINK1, Atg7, and Beclin1 in PIG1 transfected with NC-siRNA or Lonp1-siRNA. (**C**) Representative western blots and quantification of NLRP3, GSDMD-N, Cleaved caspase-1, IL-1β, and PINK1 in PIG1 after the treatment of 1.0 mM H_2_O_2_ for 24 h. (**D** & **E**) The knockdown efficiency of PINK1-siRNA in PIG1 cells assessed at the RNA level (by qRT-PCR) and protein level (by Western blot). (**F**) Representative western blots and quantification of NLPR3, GSDMD-N, Cleaved caspase-1 and IL-1β levels in PIG1 transfected with NC-siRNA or PINK1-siRNA. NC, negative control. Data are shown as mean ± SD (*n* = 3). *p < 0.05, **p < 0.01, ***p < 0.001 and ****p < 0.0001

Lonp1 is a crucial mitochondrial functional protein. Therefore, we examined its effects on mitochondria using JC-1 staining. We observed enhanced green fluorescence in Lonp1-deficient cells, reflecting mitochondrial depolarization (Fig. 3A). Meanwhile, si-Lonp1 reduced levels of PINK1 (0.69-fold; *p* = 0.0018), a key protein in the classical mitophagy pathway, and of the autophagy-associated proteins Atg7 (0.88-fold; *p* = 0.0044) and Beclin1 (0.81-fold; *p* = 0.0412) in PIG1 cells (Fig. 3B). These results suggested that Lonp1 deficiency in melanocytes may induce mitochondrial damage and suppress the mitophagy process.

We measured protein expression levels of PINK1 and the NLRP3 inflammasome pathway after H_2_O_2_ stimulation in PIG1 cells, and we observed increases in the NLRP3 pathway, while PINK1 showed a decreasing trend (0.52-fold; *p* = 0.0037) (Fig. 3C). This is consistent with the effects produced by Lonp1 knockdown. To explore the relationship between PINK1 and NLRP3 inflammasome, we exposed PIG1 cells to PINK1-siRNA (Fig. 3D, E). Western blot findings revealed that mitophagy inhibition triggers NLRP3 inflammasome activation (1.60-fold; *p* = 0.0026), thereby enhancing IL-1β expression (1.35-fold; *p* = 0.0341) (Fig. 3F). These results support our hypothesis that Lonp1 reduction impairs mitochondrial function and suppresses mitophagy initiation via downregulating PINK1 in PIG1 cells, leading to inflammasome activation and cell pyroptosis.

### Lonp1 Knockdown-induced NLRP3 Inflammasome Activation is Attenuated by PINK1 Overexpression or Mitophagy Induction In Vitro

**Fig. 4:**
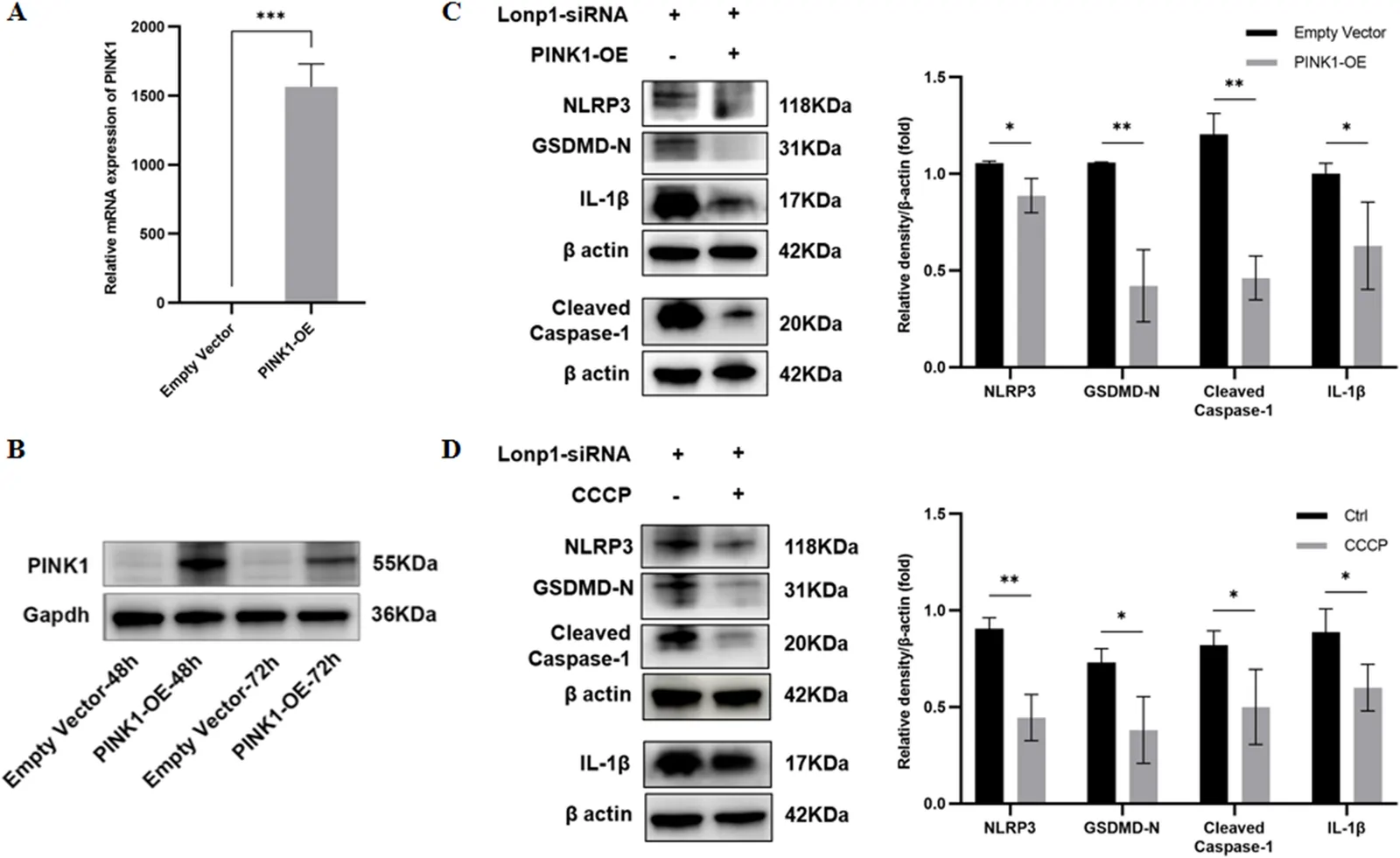
PINK1 overexpression or mitophagy induction attenuates Lonp1 knockdown-induced NLRP3 inflammasome activation (**A** & **B**) The over-expression efficiency of PINK1 in PIG1 cells on RNA and protein levels. (**C**) Representative western blots and quantification of NLRP3, GSDMD-N, cleaved caspase-1, and IL-1β in PIG1 cells transfected with Lonp1 siRNA plus either empty vector or PINK1 overexpression plasmid. (**D**) Representative western blots and quantification of NLRP3, GSDMD-N, cleaved caspase-1, and IL-1β in PIG1 cells treated with 20µM CCCP. Data are shown as mean ± SD (*n* = 3). *p < 0.05, **p < 0.01, and ***p < 0.001

To investigate the role of PINK1 in the Lonp1/NLRP3 axis, we constructed a PINK1 overexpression plasmid and confirmed its efficiency at both mRNA and protein levels (Fig. 4A, B). Subsequently, PIG1 cells were co-transfected with Lonp1-siRNA and the PINK1 overexpression plasmid. Analysis of NLRP3 inflammasome-related protein expression revealed that Lonp1 knockdown-induced hyperactivation of NLRP3 was effectively rescued by PINK1 overexpression, which decreased the protein levels of NLRP3, GSDMD-N, cleaved caspase-1, and IL-1β to 0.84-fold (*p* = 0.0300), 0.40-fold (*p* = 0.0041), 0.38-fold (*p* = 0.0012), and 0.63-fold (*p* = 0.0489) of the Lonp1-knockdown group, respectively (Fig. 4C). Furthermore, treatment with CCCP, a mitophagy inducer, similarly suppressed NLRP3 pathway activation in Lonp1-knockdown PIG1 cells, as evidenced by decreased protein levels of NLRP3, GSDMD-N, cleaved caspase-1, and IL-1β to 0.49-fold (*p* = 0.0037), 0.52-fold (*p* = 0.0321), 0.61-fold (*p* = 0.0183), and 0.68-fold (*p* = 0.0442) of the Lonp1-knockdown control, respectively (Fig. 4D). Together, these findings indicate that PINK1-mediated mitophagy may serve as a critical link between Lonp1 deficiency and NLRP3 inflammasome activation in PIG1 cells.

### Decreased Lonp1/PINK1 and Increased NLRP3 Levels are Observed in Melanocytes from the Tail Skin of the CD8⁺ T Cell–Mediated Vitiligo Mouse Model

**Fig. 5:**
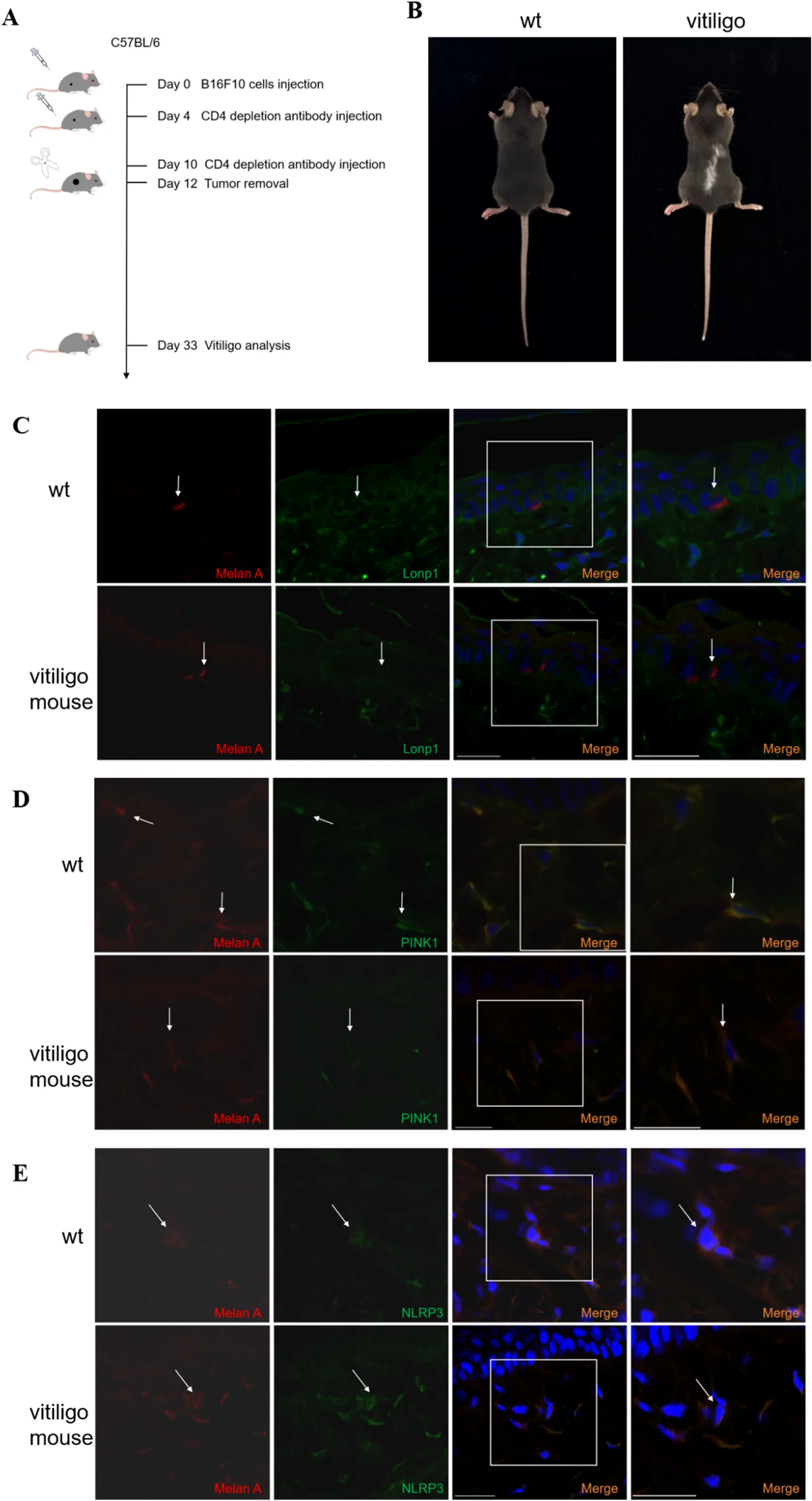
Immunofluorescence analysis of Lonp1, PINK1, and NLRP3 in melanocytes from the melanoma-associated, CD8^+^T cell–mediated vitiligo mouse model. (**A**) Schematic illustration of the mouse model induction process (melanoma-Treg-induced vitiligo). (**B**) Representative photographs of wild-type (wt) and vitiligo model mice, showing the depigmentation phenotype on the tail. (**C**) Lonp1 (green) immunofluorescence in tail skin. In vitiligo model mice, Lonp1 fluorescence intensity in melanocytes is decreased compared to wt mice. (**D**) PINK1 (green) immunofluorescence. In vitiligo model mice, PINK1 signal in melanocytes is decreased. (**E**) NLRP3 (green) immunofluorescence. In vitiligo model mice, NLRP3 signal in melanocytes is increased. Nuclei were counterstained with DAPI (blue). Melanocytes were counterstained with Melan A (red). Nuclei were counterstained with DAPI (blue). White arrows indicate melanocytes. Scale bar = 20 μm

To provide further validation of our results, a vitiligo mouse model was subsequently established via B16F10 melanoma cell inoculation in conjunction with CD4⁺ T cell depletion (Fig. 5A). Following the development of a pronounced depigmentation phenotype in the tail (Fig. 5B), skin tissues were harvested for the preparation of frozen sections. Immunofluorescence analysis revealed a reduction in Lonp1 (Fig. 5C) and PINK1 (Fig. 5D), and an increase in NLRP3 levels (Fig. 5E) in the melanocytes of the vitiligo mouse tail skin, thereby corroborating our earlier findings.

### Reduced Lonp1/PINK1 May Induce Increased NLRP3 Inflammasome in the Lesional Skin of Progressive Vitiligo Patients

**Fig. 6:**
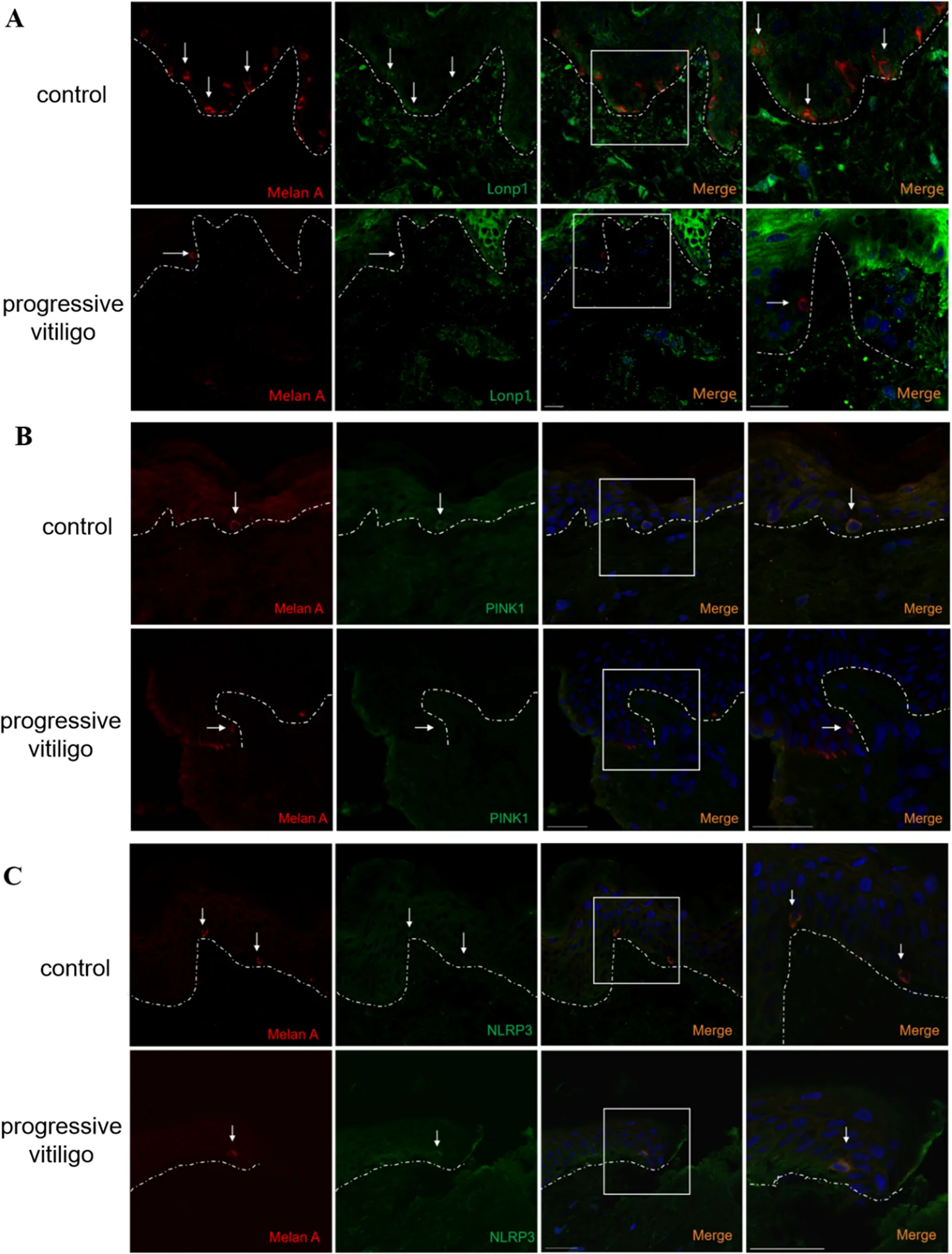
Immunofluorescence analysis of Lonp1, PINK1, and NLRP3 in skin tissues from healthy controls and patients with progressive vitiligo. (**A**) Lonp1 (green) immunofluorescence. In vitiligo patients, Lonp1 fluorescence intensity in melanocytes is decreased compared to healthy controls. (**B**) PINK1 (green) immunofluorescence. In vitiligo patients, PINK1 signal in melanocytes is decreased. (**C**) NLRP3 (green) immunofluorescence. In vitiligo patients, NLRP3 signal in melanocytes is increased. Melanocytes were counterstained with Melan A (red). Nuclei were counterstained with DAPI (blue). Scale bar = 20 μm

To investigate whether the aforementioned findings are consistent in patients with progressive vitiligo, we collected skin lesions adjacent to the depigmented areas and performed immunofluorescence staining. The results indicated decreased Lonp1 (Fig. 6A) and PINK1 (Fig. 6B) in the skin lesions of patients with progressive vitiligo. Increased NLRP3 level was also observed in the melanocytes of skin lesions (Fig. 6C). This suggests that in progressive vitiligo, the downregulation of Lonp1 induced by oxidative stress, which may lead to melanocyte pyroptosis, is one of the contributing factors in the pathogenesis of vitiligo.

## Discussion

**Fig. 7:**
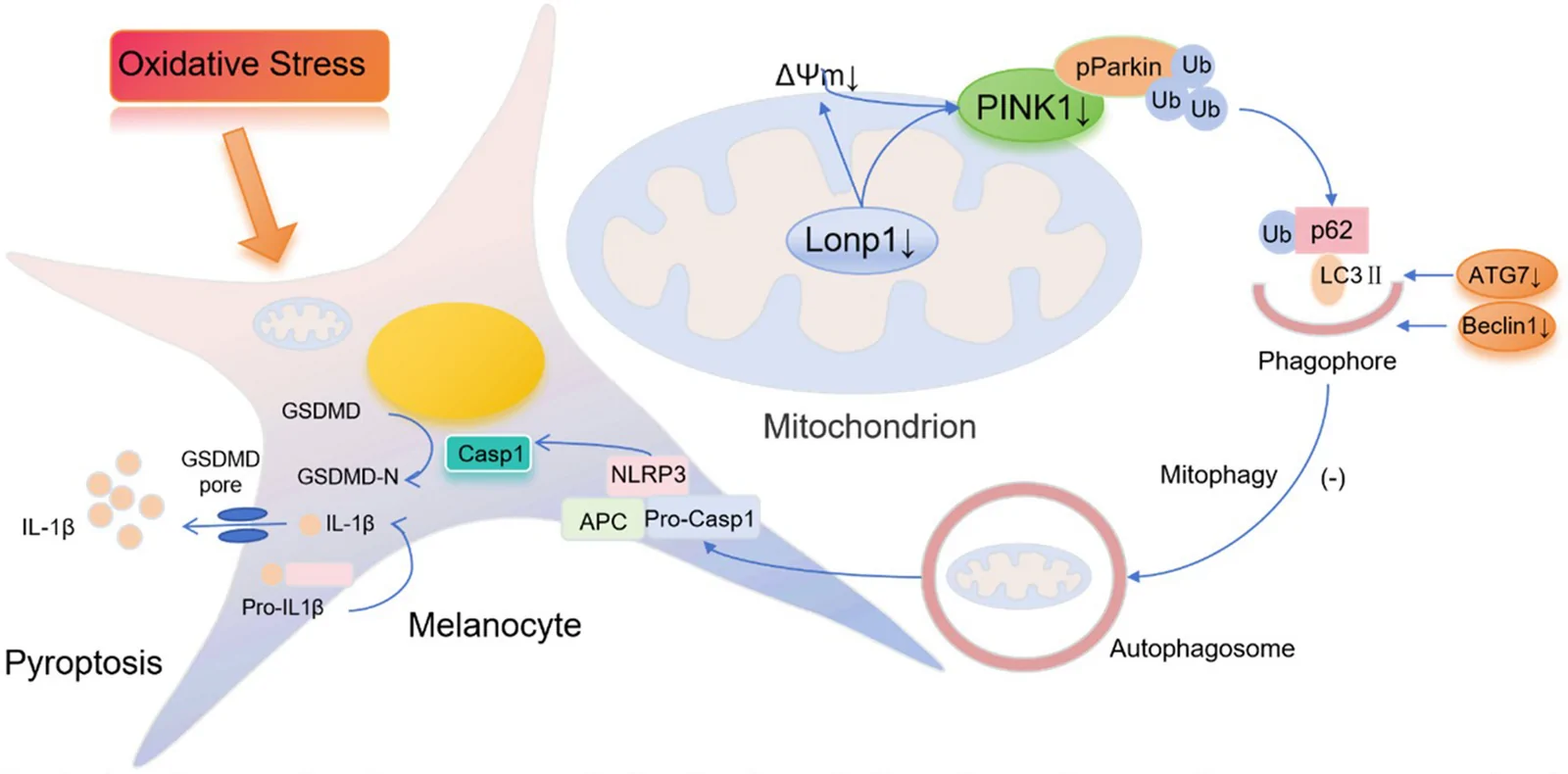
Hypothetical pathway of melanocyte regulation by Lonp1: Lonp1 regulates melanocyte pyroptosis via the Lonp1-PINK1-NLRP3/IL-1β axis in PIG1 cells under oxidative stress. The pathway of formation impairment of phagophores and autophagosomes following PINK1 downregulation in this diagram is inferred and built using findings from previous studies in mammals [23] and has not been validated in the present study

Lonp1, an abundant ATP-dependent protease of the mitochondrial matrix, works as both a protease and a molecular chaperone [11, 15]. The role of Lonp1 in maintaining functional homeostasis has been extensively studied in various cell types, such as cardiomyocytes [16], skeletal muscle cells [17], hepatocytes [18], and cancer cells [19]. It is crucial for maintaining normal mitochondrial physiological activity. It has been established that Lonp1 was overexpressed in cancer cells [20]. And mitochondrial oxidative stress selectively suppresses LONP1 and CLPP in cancer cells, thereby promoting proteotoxic stress and cell death. This is considered a potential therapeutic strategy for cancer [21]. Furthermore, Wang et al. demonstrated that in keratinocytes, Lonp1 interacts with RARRES1 to facilitate the degradation of TFAM, thereby regulating mitochondrial biogenesis. Targeting the RARRES1-LONP1/TFAM axis was shown to have significant potential for inhibiting cutaneous squamous cell carcinoma development [22]. In this study, we found that Lonp1 expression is downregulated both in lesional melanocytes from patients with progressive vitiligo and in a H_2_O_2_-induced oxidative stress cell model. Furthermore, in vitro experiments in PIG1 cells indicated that Lonp1 may regulate pyroptosis via the Lonp1-PINK1-NLRP3 axis. (Fig. 7).

Mitochondrial quality control (MQC) is an integrated network that monitors mitochondrial integrity and serves as an endogenous protective cellular program, which is essential for maintaining mitochondrial homeostasis and function. Mitophagy eliminates damaged mitochondria, thus safeguarding cells from the detrimental effects of abnormal mitochondrial accumulation [24]. PTEN-induced putative kinase 1 (PINK1) /PARKIN is the classic mitophagy initiation ubiquitin-dependent pathway. A normal mitochondrial membrane potential (MMP) facilitates PINK1 import into the inner membrane, targeting it for degradation and inactivation [25]. In contrast, MMP depolarization impedes this import, resulting in PINK1 stabilization and accumulation on the outer membrane. This accumulated PINK1 then serves as the signal to recruit and activate the E3 ubiquitin ligase Parkin, initiating mitophagy in a coordinated effort to preserve mitochondrial quality.

Although a role for Lonp1 in mitophagy is recognized, and this study has demonstrated that Lonp1 deficiency could lead to the inhibition of mitophagy in melanocytes, its reported consequences are inconsistent across different cell models. Lonp1 is involved in the dynamic and fine-tuned regulation necessary for myoblast differentiation and myotube maturation. Lonp1 acts through a multi-faceted mechanism: it triggers mitochondrial depolarization, inhibits the PINK1/Parkin pathway, and downregulates Mfn2 and Drp1, thereby preventing the mitochondrial remodeling that is critical for terminal differentiation [26]. In mouse skeletal muscle cells, loss of Lonp1 induces autophagy and causes muscle loss and weakness [17]. Furthermore, Depletion of Lonp1 in immortalized human dermal fibroblasts led to suppressed mitochondrial protein expression. Conversely, it elevated the steady-state level of the mitophagy marker PINK1 and induced concerted activation of the integrated stress response pathway [27]. However, other findings demonstrate that Lonp1 knockdown suppresses multiple malignant phenotypes, including proliferation, migration, and invasion, and induces apoptosis in cervical cancer cells [19]. This heterogeneity may reflect the distinct physiological functions of the cells studied and variations in experimental conditions.

Furthermore, this study found that Lonp1 deficiency leads to a decrease in mitochondrial membrane potential. According to the canonical model of mitophagy regulation, loss of membrane potential should result in the stabilization and accumulation of PINK1 on the outer mitochondrial membrane, which in turn phosphorylates Parkin and initiates the autophagic clearance program. However, unexpectedly, upon Lonp1 knockdown, the protein level of PINK1 did not increase but rather decreased significantly, accompanied by downregulation of the autophagy-related proteins Atg7 and Beclin-1. These findings suggest that the mitochondrial damage induced by Lonp1 deficiency does not effectively activate the canonical PINK1/Parkin-mediated mitophagy pathway, and that there may be additional cellular mechanisms suppressing this pathway.

In our study, we found that overexpression of PINK1 or treatment with a mitophagy agonist reversed the Lonp1 deficiency-mediated activation of the NLRP3 inflammasome and significantly reduced the cleavage of the downstream pyroptosis‑related protein GSDMD as well as the secretion of IL-1β, suggesting that restoring mitophagy may inhibit melanocyte pyroptosis by clearing damaged mitochondria. The importance of mitophagy dysregulation in diseases is self-evident; however, mitophagy exhibits a dual nature. On the one hand, moderate mitophagy selectively eliminates damaged or dysfunctional mitochondria, thereby limiting excessive mitochondrial ROS production and mtDNA leakage, which inhibits inflammatory signaling and maintains cell survival. In Parkinson’s disease, loss of PINK1/PRKN-mediated mitophagy leads to damaged mitochondrial accumulation and accelerated neuronal death [28]; in UVB-induced skin keratinocyte damage, impaired mitophagy causes mtDNA leakage into the cytosol, activating the cGAS-STING pathway and triggering type I interferon responses and apoptosis [29]. On the other hand, excessive or aberrant mitophagy under specific pathological conditions can also be harmful. In a sepsis model, forced restoration of mitophagy leads to immune paralysis and aggravated sepsis [30]. Moreover, in the tumor microenvironment, cancer cells upregulate mitophagy to remove therapy-induced damaged mitochondria, reduce oxidative stress, and gain therapeutic resistance, while excessive mitophagy can cause mitochondrial network fragmentation and energy crisis, triggering cell death [31]. Therefore, the role of mitophagy in diseases follows a principle that moderate activity is beneficial. Future studies should further elucidate context-specific differences in mitophagy regulatory networks to provide a theoretical basis for precise intervention.

The NOD-like receptor (NLR) family comprises four members, including NLRA, NLRB, NLRC, and NLRP, among which the NOD-like receptor protein 3 (NLRP3) has been the most extensively studied [32]. It is extensively involved in the pathological development of various inflammation-related diseases [33]. It comprises a Nod-like receptor (NLR), an apoptosis-associated speck-like protein containing a caspase recruitment domain (ASC), and a caspase-1 precursor. Their assembly and activation induce the proteolytic cleavage of cytokine precursors, resulting in the maturation and release of IL-1β and IL-18, which are potential signs of pyroptosis [34]. Prior research has shown that oxidative stress in keratinocytes promotes NLRP3 inflammasome activation and subsequent IL-1β production via regulation of TRPM2 channel function, leading to enhanced CD8⁺ T cell chemotaxis and ultimately aggravating vitiligo [35]. This highlights the important connection between inflammasome activation and the immune processes underlying vitiligo. Studies have established a link between mitophagy/autophagy and the regulation of inflammasome activation and pyroptosis [36, 37]. In Parkinson’s disease, PARK2/PINK1 deficiency in primary microglia heightened NLRP3 inflammasome activity and aggravated neuroinflammation [28]. In neuroinflammation caused by LPS, overexpressing TRIM45 promotes NLRP3 activation in an autophagy-dependent manner based on its E3 ubiquitin ligase activity [38]. A recent study revealed that NLRP3 in vitiligo melanocytes becomes hyperactivated due to impaired autophagic degradation, driving melanocyte pyroptosis—a process mediated by downregulated E3 ligase β-TrCP1 [39]. Our study revealed that mitochondrial autophagy can also regulate the activation of the NLRP3 inflammatory pathway in melanocytes. The difference is that mitophagy focuses on removing the damaged mitochondrial source to reduce NLRP3 activation signals, whereas selective autophagy directly targets the NLRP3 protein for degradation. These two mechanisms may play a synergistic role in the pyroptosis of melanocytes in vitiligo. This provides important insights: beyond keratinocytes, NLRP3 activation in melanocytes is also closely linked to vitiligo pathogenesis.

Overall, our study identifies Lonp1 as a pivotal protein for maintaining mitochondrial function and oxidative stress defense in melanocytes. Deficiency in Lonp1 may impair mitophagy, thereby disrupting mitochondrial homeostasis and triggering the NLRP3 inflammasome pathway, which induces pyroptosis in melanocytes.

## Materials and Methods

### Single-Cell Sequencing Data Analysis

In this study, single-cell RNA sequencing data of skin tissues from vitiligo patients and healthy controls were obtained from the Genome Sequence Archive (GSA) database (PRJCA006797). After quality control and normalization of the raw data, an unsupervised clustering algorithm was applied for cell grouping. Subsequently, melanocyte populations were accurately identified and extracted based on known cell type-specific marker genes. On this basis, melanocytes were divided into three groups according to sample origin: healthy control, stable patients, and progressive patients. The expression levels of the Lonp1 gene in melanocytes were then compared among the three groups.

### Patients and Skin Samples

Normal human skin samples for this study were sourced from the biobank of the Institute of Dermatology, Chinese Academy of Medical Sciences, and Peking Union Medical College. Lesional skin specimens were acquired from patients diagnosed with progressive vitiligo at our institution. The study protocol received ethical approval [Approval No.: 2025-KY-038], and all procedures involving human participants were conducted after obtaining informed consent and in adherence to the Declaration of Helsinki.

### Cell Culture and Treatment

Two immortalized human melanocyte cell lines, PIG1 (BFN60808828) and PIG3V (BFN60810796) cells were obtained from Bluefbio (Shanghai) Biotechnology Development Co., Ltd. The PIG1 cell line was established from normal human neonatal foreskin melanocytes, while the PIG3V cell line was derived from the perilesional skin of a patient with vitiligo. Melanocyte cell lines were cultured in Sodium Pyruvate-free DMEM with 10% Fetal Bovine Serum (FBS, Gibco, Brazil) and 1% penicillin/streptomycin (NCM Biotech, C100C5) at 37 ℃ in an atmosphere containing 5% CO_2_. Hydrogen peroxide (H_2_O_2_, 3%, Macklin) at a concentration of 1 mmol/L was used to induce a cellular oxidative stress model.

### siRNA Transfection

siRNAs against Lonp1/PINK1 and transfection reagent were purchased from GenePharma (Shanghai, China). First, the siRNA was mixed with the transfection buffer. Then this siRNA premix was combined with the siRNA-mate plus transfection reagent, and the resulting mixture was added to the cell culture medium. After incubation for 24 to 72 hours, RNA or protein samples were collected for analysis. The following sequences were used: Lonp1-siRNA1: 5’-GUCGGCGUCUUUCUAAAGATT-3’, Lonp1-siRNA2: 5’-GGGACAUCAUUGCCUUGAATT-3’, PINK1-siRNA: 5′-GAGUAGCCGCAAAUGUGCUUCAUCU-3′ and negative control siRNA: 5’-UUCUCCGAACGUGUCACGUTT-3’.

### Plasmid Transfection

For Lonp1 knockdown and subsequent PINK1 overexpression, PIG1 cells were first transfected with Lonp1-specific siRNA (or control siRNA). Twelve hours post-transfection, the culture medium was replaced with fresh serum-free medium. For PINK1 overexpression, cells were then transfected with a PINK1 overexpression plasmid or empty vector control using Lipofectamine 3000 reagent (Thermo Fisher Scientific), following the manufacturer’s protocol. Cells were harvested for further analysis at 48 h after plasmid transfection.

### Cell Viability Assay

Cell Counting Kit-8 was purchased from Vazyme (Nanjing, China). The PIG1 cells (siRNA transfection) in the 96-well plate were supplemented with CCK-8 solution at 10% of the culture medium volume. After a 1-hour incubation at 37 °C under 5% CO₂, the optical density at 450 nm was determined using a microplate reader.

### Immunofluorescence Staining

Then the slides were incubated overnight at 4 ℃ with primary antibodies against MelanA (Abcam, ab283819, 1:1000), Lonp1 (Proteintech, 66043-1-Ig, 1:100), and NLRP3 (Abcam, ab283819, 1:50). Fluorophore-conjugated secondary antibodies were added and incubated for 1 h at room temperature. Images were captured using the confocal Microscope.

### Flow Cytometry Detection of Cell Death

PIG1 cell death was tested by Annexin V-FITC/7-AAD Apoptosis Kit (Elabscience, Wuhan, China). Following a 72-hour siRNA treatment, PIG1 cells were digested with 0.25% trypsin-EDTA, collected by centrifugation, and washed. 10^5^~10^6^ cells were resuspended in 1X Annexin V binding buffer. Subsequently, 2.5 µL of Annexin V-FITC and 2.5 µL of 7-AAD reagent were added to the cell suspension, which was then incubated at room temperature for 15 min, protected from light. Finally, 400 µL of binding buffer was added to the sample and mixed, and analysis was performed using a flow cytometer.

### JC-1 Staining

The mitochondrial membrane potential in PIG1 cells was determined by the JC-1 fluorescent probe Kit (MedChemExpress, Shanghai, China). After being treated with Lonp1-siRNA for 72 h, PIG1 cells were incubated with complete medium containing 2 µM JC-1 for 15 min. Subsequently, the supernatant was removed, and the cells were washed three times with 1X PBS. After the final wash, the cells were covered with 1X PBS, and the intracellular green and red fluorescence intensities were observed under a fluorescence microscope.

### RNA Isolation, Reverse-Transcription and Quantitative PCR (qPCR)

Total RNA was extracted from PIG1 cells with FreeZol Reagent (Vazyme, Nanjing, China). The RNA was reverse transcribed into cDNA using HiScript III RT SuperMix for qPCR (Vazyme, Nanjing, China). qPCR was performed with SYBR Green Master Mix (Vazyme, Nanjing, China) to detect the mRNA levels of Lonp1 and PINK1. GAPDH was used as an internal control. The primers used in this experiment were synthesized by Sangon Biotech (Shanghai) Co., Ltd., and the primer sequences are as follows. Lonp1: 5’-CGATGTGTTTCCGCACCTG-3’, 3’-AAGACGCCGACATAAGGCTG-5’; PINK1༚5’-CCCTCACCCCAACATCATCC-3’, 3’-ATAACGAGGAACAGCGTCCG-5’; GAPDH: 5’-GATTCCACCCATGGCAAATTC-3’, 3’-CTGGAAGATGGTGATGGGATT-5’.

### Western Blot Analysis

Total cellular protein was extracted using RIPA Lysis Buffer, followed by protein concentration determination via the BCA assay. Subsequently, 30 µg of protein per sample was loaded into each well of a 4–12% Precast Protein Gel (Bis-Tris, Smart-lifesciences) and electrophoretically separated, after which the proteins were transferred onto a PVDF membrane. Then the PVDF membranes were blocked with 5% skim milk for 1.5 h at room temperature and incubated with primary antibodies (Lonp1 (Proteintech, 66043-1-Ig), NLRP3 (Abcam, ab263899), GSDMD-N (Abcam, ab215203), cleaved Caspase-1 (CST, D57A2), PINK1 (CST, 6946T), Beclin-1 (Starter, S0B0739), Atg7 (Starter, S0B0424), IL-1β (CST, 12703T), β-Tubulin (Abbkine, ABL1030), Gapdh (Starter, S0B0261), β-actin (Proteintech, 66009-1-Ig)).

### Animal Model

The vitiligo mouse model was established following a protocol described in a previous study [40]. We purchased 8-week-old female C57BL6 mice from GemPharmatech LLC. and injected intradermally 2 × 10^5^ B16F10 melanoma cells on their dorsal skin at day 0. Then, an anti-CD4 depletion antibody (10 µg/g body weight) was injected intraperitoneally on days 4 and 10. On day 12, the dorsal tumors were surgically resected.

### Statistical Analysis

All quantitative data are expressed as mean ± SD. Data analyses were performed using GraphPad Prism (version 9.5). For comparisons between two groups, an unpaired, two-tailed Student’s t-test was applied. Comparisons among multiple groups were conducted using one-way ANOVA followed by Tukey’s honest significant difference (HSD) post hoc test. p-value<0.05 was considered statistically significant, and significance levels are denoted as ns (not significant), *p < 0.05, **p < 0.01, and ***p < 0.001.

## Data Availability

The single-cell RNA-sequencing data used in this work was deposited in the GSA with accession number PRJCA006797 (https://ngdc.cncb.ac.cn/bioproject/browse/PRJCA006797).
